# Beamline commissioning for microscopic measurements with ultraviolet and soft X-ray beam at the upgraded beamline BL-13B of the Photon Factory

**DOI:** 10.1107/S160057752200090X

**Published:** 2022-02-16

**Authors:** Kenichi Ozawa, Yoshihiro Aiura, Daisuke Wakabayashi, Hirokazu Tanaka, Takashi Kikuchi, Akio Toyoshima, Kazuhiko Mase

**Affiliations:** a Institute of Materials Structure Science, High Energy Accelerator Research Organization (KEK), Tsukuba, Ibaraki 305-0801, Japan; b Department of Materials Structure Science, SOKENDAI (The Graduate University for Advanced Studies), Tsukuba, Ibaraki 305-0801, Japan; c Department of Chemistry, Tokyo Institute of Technology, Meguro, Tokyo 152-8551, Japan; d Research Institute for Advanced Electronics and Photonics, National Institute of Advanced Industrial Science and Technology (AIST), Tsukuba, Ibaraki 308-8568, Japan

**Keywords:** VUV-SX beamline, microscopic measurement, X-ray photoelectron spectroscopy, X-ray absorption spectroscopy, angle-resolved photoelectron spectroscopy

## Abstract

Beamline 13 of the Photon Factory has been in operation since 2010 as a vacuum ultraviolet and soft X-ray undulator beamline for X-ray photoelectron spectroscopy (XPS), X-ray absorption spectroscopy (XAS), and angle-resolved photoelectron spectroscopy (ARPES) experiments. The beamline and the end-station at branch B have been recently upgraded, enabling microscopic XPS, XAS, and ARPES measurements to be performed. In 2015, a planar undulator insertion device was replaced with an APPLE-II (advanced planar polarized light emitter II) undulator. This replacement allows use of linear, circular, and elliptical polarized light between 48 and 2000 eV with photon intensities of 10^9^–10^13^ photons s^−1^. For microscopic measurements, a toroidal post-mirror was renewed to have more focused beam with profile sizes of 78 µm (horizontal) × 15 µm (vertical) and 84 µm × 11 µm at photon energies of 100 and 400 eV, respectively. A high-precision sample manipulator composed of an *XYZ* translator, a rotary feedthrough, and a newly developed goniometer, which is essential for microscopic measurements, has been used to control a sample specimen in six degrees of freedom, *i.e.* translation in the *X*, *Y*, and *Z* directions and rotation in the polar, azimuthal, and tilt directions. To demonstrate the performance of the focused beams, one- and two-dimensional XPS and XAS scan measurements of a copper grid have been performed. It was indicated from analysis of XPS and XAS intensity maps that the actual spatial resolution can be determined by the beam size.

## Abstract 2

Beamline BL-13B of the Photon Factory and the end-station have been upgraded, enabling microscopic XPS, XAS, and ARPES measurements with a spatial resolution that is comparable with the size of the focused beam. Beam profile evaluation and experimental demonstration of microscopic measurements are presented.

## 1. Introduction

Beamline 13A (BL-13A) of the Photon Factory (PF), High Energy Accelerator Research Organization (KEK), was commissioned in January 2010 as a vacuum ultraviolet and soft X-ray (VUV-SX) undulator beamline (Mase *et al.*, 2010 ▸; Toyoshima *et al.*, 2011 ▸, 2013 ▸). The beamline is an entrance slitless type and is composed of a focusing pre-mirror (M1), a plane mirror (M2), varied-line-spacing gratings (VLSGs), an exit slit, and focusing post-mirrors (M3). A variable-included-angle Monk–Gillieson-type monochromator with the VLSG (Amemiya & Ohta, 2004 ▸) is employed to monochromatize the synchrotron radiation. Two VLSGs with line densities of 300 and 1000 lines mm^−1^ covers photon energies (*h*ν) in the VUV-SX region (Toyoshima *et al.*, 2013 ▸).

In 2013, a branch beamline (BL-13B) was constructed to meet the increasing demand for beam time and to use the beam time more efficiently (Toyoshima *et al.*, 2015 ▸). Gold-, chromium-, and nickel-coated plane mirrors (Mp) have been installed on the downstream side of the monochromator at a grazing angle of 2° to guide the beam to BL-13B. The components of the branch part were an exit slit and two toroidal M3 mirrors, whose focus positions were 2 and 6 m downstream from the M3 position. Three photoelectron spectroscopy apparatuses, which were initially operated on BL-13A, were transferred to BL-13B. Apparatus equipped with an SES200 electron energy analyzer (Gamma Data/Scienta) (Toyoshima *et al.*, 2013 ▸), called the SES200 system hereafter, was placed at the 2 m focus position and was used for X-ray photoelectron spectroscopy (XPS), angle-resolved photoelectron spectroscopy (ARPES), and X-ray absorption spectroscopy (XAS) experiments. At the 6 m focus position, a near-ambient-pressure XPS (NAP-XPS) system equipped with an EA125HP analyzer for high-pressure experiments (Omicron) (Toyoshima & Kondoh, 2015 ▸) was installed. The third apparatus was for low-temperature XPS measurements (Koitaya *et al.*, 2013 ▸) and was installed between the SES200 and NAP-XPS systems. These measurement systems at BL-13B substantially contributed to research in catalysis science (Isegawa *et al.*, 2021 ▸; Kim *et al.*, 2020*a* ▸; Ozawa *et al.*, 2018 ▸; Toyoshima & Kondoh, 2015 ▸), surface science (Aiura *et al.*, 2019 ▸; Gunjo *et al.*, 2021 ▸; Koitaya *et al.*, 2013 ▸, Koitaya *et al.*, 2021 ▸), and industrial materials science (Jing *et al.*, 2020 ▸; Ozawa *et al.*, 2013 ▸; Toyoshima *et al.*, 2021 ▸).

In this paper, we outline several improvements and changes made on BL-13 after our last report in 2015 (Toyoshima *et al.*, 2015 ▸) and describe the current status of the beamline and the SES200 system for microscopic XPS, XAS, and ARPES experiments.

## 2. Insertion device upgrade

BL-13 was commissioned in January 2010. The insertion device was a 2.5 m-long multipole wiggler (MPW#13), which had 27 magnetic poles with a period length of 18 cm (Sasaki *et al.*, 1989 ▸). The insertion device provided horizontal linear polarized radiation with a brilliance from 0.5 × 10^15^ to 2 × 10^15^ photons s^−1^ mm^−2^ mrad^−2^ (0.1% bandwidth)^−1^ in the *h*ν range between 10 and 900 eV in an undulator mode. In February 2015, this insertion device was replaced by an APPLE-II (advanced planar polarized light emitter II) type elliptically polarizing undulator (EPU), U#13. The period length and the periodicity number of the APPLE-II EPU are 7.6 cm and 47, respectively (Tsuchiya, 2015 ▸; Tsuchiya *et al.*, 2016 ▸). The undulator enables linear polarized radiation (horizontal and vertical), circular polarized radiation (left and right), and elliptical polarized radiation (left and right) to be generated. The brilliance is 2 × 10^16^ to 1 × 10^18^ photons s^−1^ mm^−2^ mrad^−2^ (0.1% bandwidth)^−1^ between 50 and 2000 eV in the case of horizontal linear polarized light (Tsuchiya, 2015 ▸).

**Figure 1:**
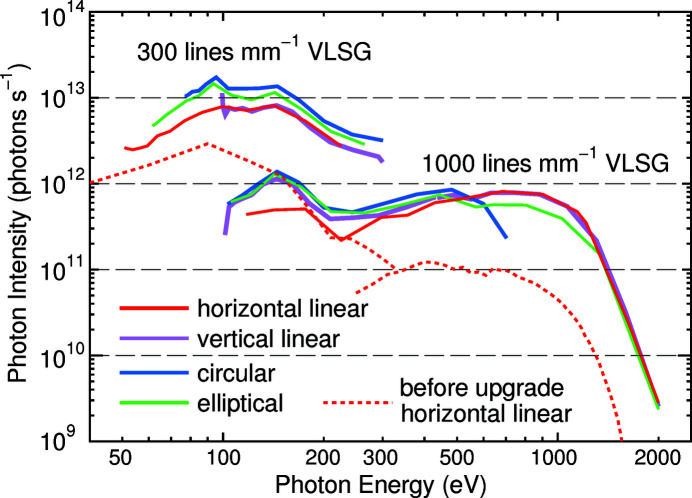
Photon intensity of BL-13A in the photon-energy region between 50 and 2000 eV after installing the APPLE-II EPU. The photon intensities were converted to equivalent values for a ring current of 450 mA. The VLSGs with line densities of 300 and 1000 lines mm^−1^ cover photon energies between 48 and 300 eV and between 100 and 2000 eV, respectively. The photon intensity variation before the insertion-device upgrade is shown by dotted lines. The exit-slit width was 100 µm.

Fig. 1 ▸ shows changes of photon intensities of various polarized light as a function of *h*ν. We measured the intensity at the downstream side of the M3 mirror in BL-13A using an Si photodiode (AXUV100, International Radiation Detectors). The exit-slit width was fixed at 100 µm, which gave photon energy resolutions (*E*/Δ*E*) ranging from 6500 (*h*ν = 64.1 eV) to 5000 (401.1 eV) to 4000 (867.1 eV) (Toyoshima *et al.*, 2013 ▸). The measurements were performed in storage mode, and the measured photon intensities were converted to equivalent values for a ring current of 450 mA, a typical current in the top-up operation of the PF.

The photon-energy ranges available at BL-13 are 48–2000 eV for horizontal linear polarized light, 102–2000 eV for vertical linear polarized light, 74–700 eV for left- and right-circular polarized light, and 59–2000 eV for left- and right-elliptical polarized light. The photon intensity depends slightly on the polarization of the light, and it surpasses 1 × 10^13^ photons s^−1^ between *h*ν = 80 and 160 eV for the circular and elliptical polarized light when the 300 lines mm^−1^ VLSG is used. The intensity of the horizontal and vertical linear polarized light is slightly lower than the circular and elliptical polarized light, but it is (2–8) × 10^12^ photons s^−1^ between 50 and 300 eV. Since the intensity of the horizontal linear polarized light for the 300 lines mm^−1^ VLSG was (0.1–3) × 10^12^ photons s^−1^ in the same *h*ν region before the upgrade of the insertion device, see the dotted line in Fig. 1 ▸ (Toyoshima *et al.*, 2013 ▸, 2015 ▸), the APPLE-II EPU allows us to use considerably intense light. In the higher *h*ν region for which the 1000 lines mm^−1^ VLSG covers, the upgrade is much more beneficial because photon intensities larger than 1 × 10^11^ photons s^−1^ are provided up to 1400 eV for light with linear and elliptical polarization, while such an intense light was available only between 300 and 700 eV before the upgrade (Toyoshima *et al.*, 2013 ▸, 2015 ▸). At *h*ν = 2000 eV, the photon intensity is approximately 3 × 10^9^ photons s^−1^, which is still enough to perform XPS measurements.

Although the lowest energy available for the undulator beam is 48 eV at BL-13, the photon energy can be tuned down to 30 eV. However, such light is only provided by a wiggler mode and, thus, the photon intensity is low and the light polarization is limited to a horizontal linear one. Nevertheless, the low intensity and low photon energy are advantageous for some materials that are fragile to intense light. A photoemission study of organic semiconductors has indeed been carried out at BL-13B by using the 30 eV light (Gunjo *et al.*, 2021 ▸)

It should be noted that the photon intensity is affected by the Mp mirror, and thus the photon intensity at BL-13B is modified from those shown in Fig. 1 ▸. It was estimated that, using the gold-coated Mp mirror, the photon intensity was lowered by approximately 30% between 300 and 1000 eV, whereas such an intensity decrease was minimal at photon energies below 300 eV (Toyoshima *et al.*, 2013 ▸). Thus, highly intense photons are also available at BL-13B.

## 3. Upgrade for microscopic measurements

Microscopic measurements utilizing VUV-SX synchrotron radiation have attracted much attention in the last decades especially in the research field of materials science. This situation coincides with the construction and the operation of third-generation 3 GeV synchrotron radiation facilities in the 2000s. In these facilities, microscopic and nanoscopic (micro/nano)-ARPES and micro/nano-XPS have been extensively conducted to investigate local electronic structures or local chemical states of condensed materials with micro- and nanoscale dimensions (Bannies *et al.*, 2021 ▸; Ernandes *et al.*, 2021 ▸; Kim *et al.*, 2020*b* ▸; Noguchi *et al.*, 2021 ▸).

Although the PF is a second-generation 2.5 GeV ring, BL-13 can provide high photon intensities (Fig. 1 ▸) that are not comparable with but are close to the intensities of beamlines in the state-of-the-art 3 GeV facilities. Therefore, we upgraded the SES200 system to enable microscopic ARPES, XPS, and XAS measurements of various functional materials using intense VUV-SX light. Two elements needed to be developed for the realization of microscopic measurements: one to make precise control of the sample position at the micrometre level and the other to focus the photon beam to micrometre size.

### 3.1. Precise sample manipulation

For a precise control of the sample position, a high-precision *XYZ* translator was developed with travel distances of ±15 mm and 260 mm for the *X*/*Y* axes and the *Z* axis, respectively (Aiura *et al.*, 2020 ▸). The translator consists of a mounting ConFlat (CF) flange with an outer diameter (OD) of 203 mm, a traveling CF flange with a 152 mm OD, and five linear drive units, each of which consisting of a linear actuator and a stepping motor. Two linear drive units are used for the *X*- and *Y*-axis motions, and the *Z*-axis motion is controlled by the remaining three, which run synchronously to move the traveling flange without an irregular fluctuation. An absolute optical encoder system (RESOLUTE, Renishaw) is attached to each axis and determines the position with an accuracy of 100 nm or less (a sub-divisional error of the encoder is ±40 nm). The reading resolution of the position is 1 nm. The encoder is also used as a software limit switch. The encoders can compensate for the mechanical errors of the linear actuators so that sample position control with submicrometre accuracy is possible. A study by Aiura *et al.* (2020 ▸) gives more details of the high-precision *XYZ* translator.

**Figure 2:**
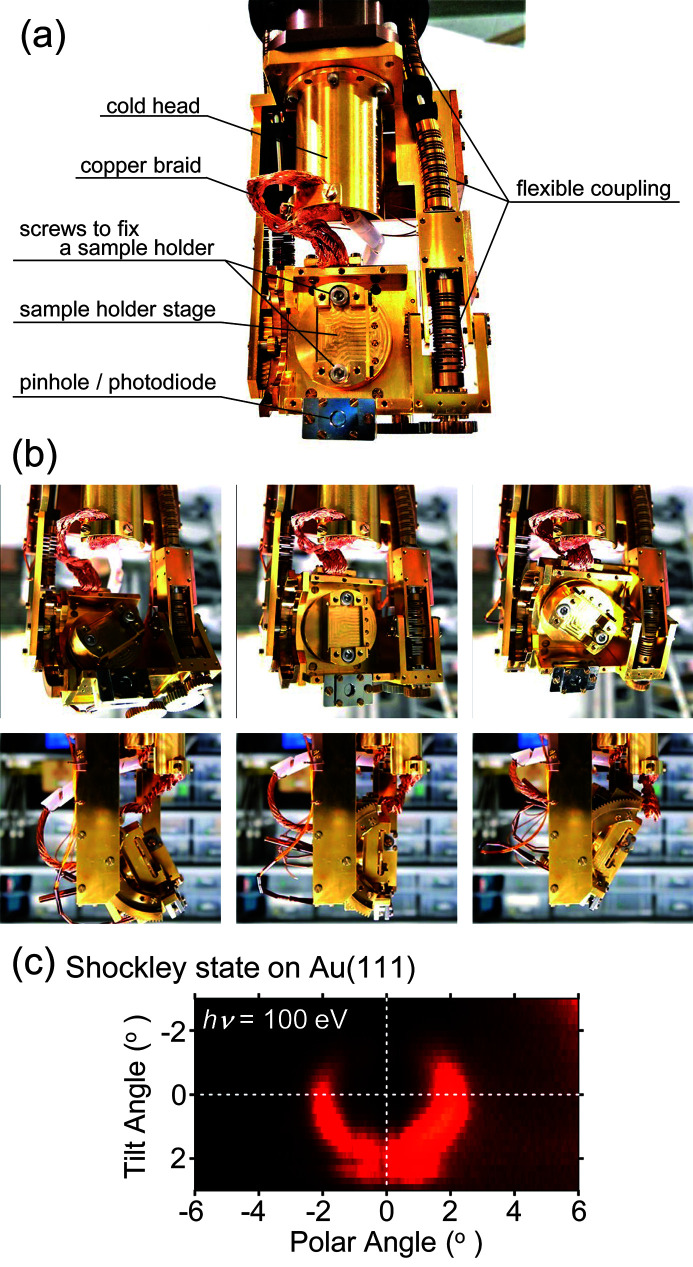
(*a*) Front image of the two-axis cryogenic sample goniometer (iGONIO-FC). (*b*) Front and side views (upper and lower images, respectively) of azimuthal and tilt rotations of the sample-holder stage. (*c*) A Fermi surface cut of Au(111) measured by right-circular polarization light at *h*ν = 100 eV (300 lines mm^−1^ VLSG). A center circle corresponds to the Shockley state, which is visualized by integrating the photoemission intensity between −0.05 and +0.05 eV in binding energy. A series of ARPES spectra were acquired by an angular mode of the SES200 analyzer with an effective acceptance angle of about ±6° (in the polar angle direction), and the tilt angle was scanned from −3.0° to +3.0° with a 0.20° step. The exit-slit width was 30 µm, and the total energy resolution was set at 0.11 eV.

For precise control of the sample rotation, we used a high-precision rotary feedthrough (iRS152, Vacuum & Optical Instruments) (Aiura & Kitano, 2012 ▸) and a newly developed two-axis cryogenic sample goniometer (iGONIO-FC, R-DEC) [Fig. 2 ▸(*a*)]. By incorporating a robust integrated-inner/outer-ring-type crossed roller bearing (RUV, THK) developed specifically for the rotary feedthrough, iRS152 realizes the rotary accuracy that considerably surpasses the conventional model (VRS152, Vacuum & Optical Instruments). An optical rotary encoder system with a reading resolution of about 0.0003 arcsec (RESOLUTE, Renishaw) was installed on the rotary feedthrough, and this enables control of the polar angle of the sample with a system accuracy of ±0.0004° (or ±1.43 arcsec) (Aiura & Kitano, 2012 ▸). The sample goniometer controls azimuthal and tilt rotation independently using two stepping motors [Fig. 2 ▸(*b*)]. The maximum available angles of azimuthal and tilt rotation are ±180° and ±45°, respectively, where the tilt angle of 0° corresponds to the direction where the sample-holder stage faces the front. It is noteworthy that the movable range of the azimuthal rotation is limited by the copper braids used for cooling, whereas the tilt rotation is mainly restricted by the mechanical design.

A capability of the sample goniometer for ARPES measurements was assessed by measuring a Fermi surface of an Au(111) surface. A series of ARPES spectra in the valence band region were acquired at room temperature by moving the tilt angle of the goniometer from −3.0° to +3.0° with a step of 0.20°, while the azimuthal and polar angles were fixed. The photon energy was 100 eV (300 lines mm^−1^ VLSG) with right-circular polarization. Fig. 2 ▸(*c*) shows a Fermi surface around the center of the surface Brillouin zone of the Au(111) surface. The center circle corresponds to the Shockley state, which is typical for the (111) surface of noble metals. Intensity anisotropy of the state is obvious, and its origin is proposed to be related to the *d* orbital contribution to the final state band of Au, *i.e.* the failure of the free-electron final state approximation (Mulazzi *et al.*, 2009 ▸). ARPES studies are also possible using the upgraded sample manipulator.

### 3.2. Focusing of the photon beam

**Figure 3:**
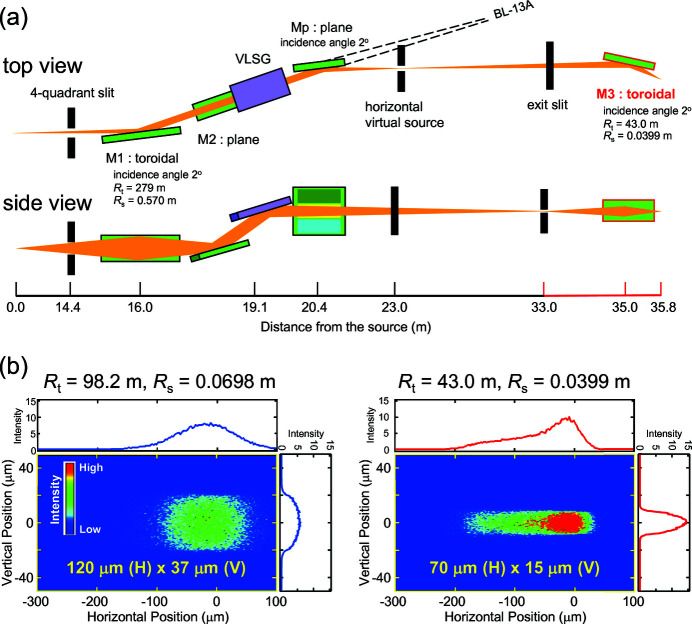
(*a*) Schematic optical layout of BL-13B. (*b*) Spot sizes at focal positions for the two toroidal focusing post-mirrors estimated by ray-tracing simulations. A photon energy of 100 eV, VLSG line density of 300 lines mm^−1^, and exit-slit width of 40 µm were assumed. Integrated intensities along the vertical and horizontal axes are shown on the upper and right sides of each panel, respectively. The origin of the horizontal vertical axes corresponds to the maximum positions of the integrated peaks.

Regarding focusing of the photon beam, we replaced the toroidal post-mirror (M3) that had tangential and sagittal radii of *R*_t_ = 98.2 m and *R*_s_ = 0.0698 m, respectively, with one having *R*_t_ = 43.0 m and *R*_s_ = 0.0399 m. Since the incidence angle of the beam to M3 is 2° and the distances between the horizontal virtual source and M3 and between the exit slit and M3 are 12.0 and 2.0 m, respectively, the new toroidal mirror gives both horizontal and vertical focal points at 0.80 m downstream from M3 [Fig. 3 ▸(*a*)], which is 1.2 m closer to M3 compared with the original one (Mase *et al.*, 2010 ▸). Fig. 3 ▸(*b*) shows simulated beam profiles at the focal positions using the *ShadowOui* ray-tracing tool (Rebuffi & Sánchez del Río, 2016 ▸). A photon energy of 100 eV, VLSG line density of 300 lines mm^−1^, and exit-slit width of 40 µm were assumed for calculating the beam profiles. A beam size of 120 µm (horizontal) × 37 µm (vertical) full width at half-maximum (FWHM) was estimated for the original M3 toroidal mirror setup with *R*_t_ = 98.2 m and *R*_s_ = 0.0698 m. By using the upgraded mirror setup with *R*_t_ = 43.0 m and *R*_s_ = 0.0399 m, it is expected to be reduced to 70 µm (H) × 15 µm (V). It is noted that a large tailing in the horizontal direction is caused by spherical aberration. According to our simulation, the beam size is estimated to be reduced to 58% (H) and 41% (V) by upgrading the M3 mirror.

## 4. Beam profile assessment

**Figure 4:**
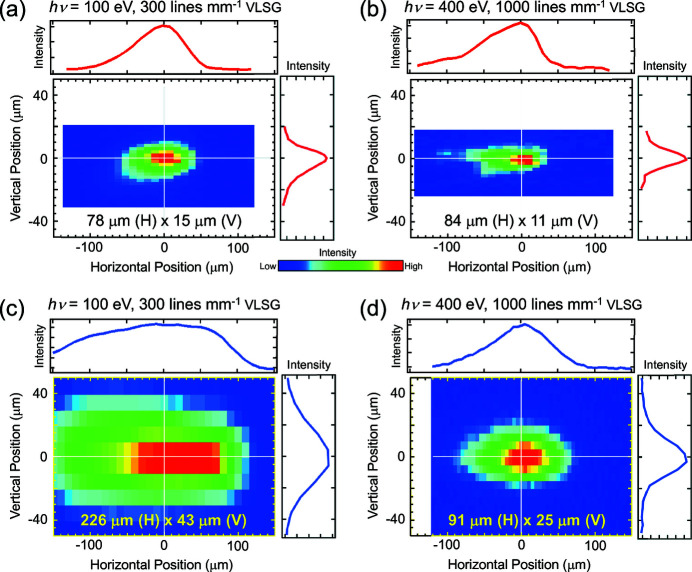
Comparison of experimentally determined profiles of the horizontal linear polarized beam (*a*, *b*) after and (*c*, *d*) before upgrading the M3 mirror. The photon energies were 100 eV (*a*, *c*) and 400 eV (*b*, *d*) using the 300 and 1000 lines mm^−1^ VLSGs, respectively. The exit-slit width was 30 µm for all measurements. The beam profiles are the images seen from the upstream side. Integrated intensities along the vertical and horizontal axes are shown on the upper and right sides of each panel, respectively.

Beam profiles of the upgraded BL-13B were assessed by measuring the spatial distribution of photocurrents. The photocurrents induced by the light passing through a pinhole with a diameter of 2 µm were detected by a Si photodiode (XUV-005, OSI Optoelectronics), which was mounted at the tip of the sample goniometer [Fig. 2 ▸(*a*)]. The light was injected from the normal direction. The results for *h*ν = 100 and 400 eV with horizontal linear polarization are shown in Figs. 4 ▸(*a*) and 4 ▸(*b*), respectively. For comparison, the beam profiles before upgrading of the M3 mirror are shown in Figs. 4 ▸(*c*) and 4 ▸(*d*) (Aiura *et al.*, 2020 ▸). For the beam at *h*ν = 100 eV with the 300 lines mm^−1^ VLSG, the beam size was 78 µm (H) × 15 µm (V), which was about one-third of the original size [226 µm (H) × 43 µm (V)]. The actual spot size after the upgrade is comparable with that predicted by the ray-tracing simulation under the same conditions except for the exit slit size [70 µm (H) × 15 µm (V), Fig. 3 ▸(*b*)]. For the beam at *h*ν = 400 eV with the 1000 lines mm^−1^ VLSG, on the contrary, the beam size was significantly reduced along the vertical direction (11 µm), less than half compared with the original one, while it is only slightly reduced along the horizontal direction (84 µm). Since the M3 mirror has a vertical magnification ratio of 1:0.4 [Fig. 3 ▸(*a*)], the observed vertical size of 11 µm is nearly identical to the expected size of 12 µm at the exit slit of 30 µm. Tailing of the beam spots by the spherical aberration was recognized in the horizontal direction for all observed beams.

**Figure 5:**
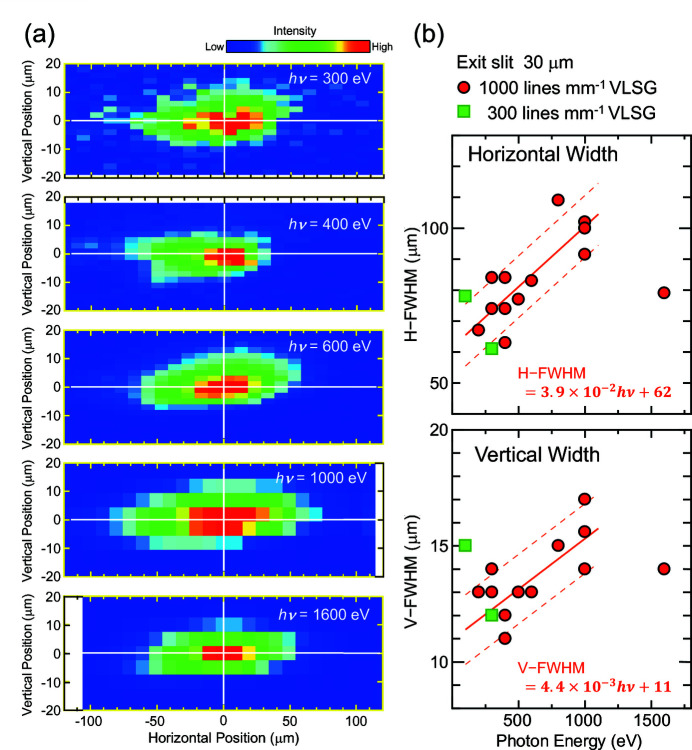
(*a*) Photon energy dependence of the beam profile of the horizontal linear polarized light (1000 lines mm^−1^ VLSG and exit-slit width of 30 µm). The beam profiles correspond to the image seen from the upstream side. (*b*) Plots of FWHMs of beam spots in the vertical and horizontal directions against photon energy. The FWHMs of beam spots using the 300 and 1000 lines mm^−1^ VLSGs are shown by squares and circles, respectively. Solid linear lines are results of the least-squares fitting of the circles between 200 and 1000 eV with the 1000 lines mm^−1^ VLSG, and dashed lines correspond to lines shifted by ±10 µm (horizontal) and ±1.5 µm (vertical).

As already pointed out in our previous study (Aiura *et al.*, 2020 ▸), the actual beam size depends on the photon energy. Fig. 5 ▸ shows an *h*ν-dependence of the beam profiles for horizontal linear polarized light. Here, the 1000 lines mm^−1^ VLSG and an exit-slit width of 30 µm were used to measure the profiles. Although the scatter of the plotted points is large, FWHMs of the beam profiles in the vertical (V-FWHM) and horizontal (H-FWHM) directions tend to increase monotonically with photon energy up to 1000 eV, as shown in Fig. 5 ▸(*b*). From the observed *h*ν-dependence of the beam widths, the V-FWHM and H-FWHM values between 200 and 1000 eV are given by the following empirical equations: V-FWHM = 4.4 × 10^−3^ *h*ν + (11 ± 1.5) and H-FWHM = 3.9 × 10^−2^ *h*ν + (62 ± 10), where the units of the FWHMs and *h*ν are µm and eV, respectively. Interestingly, the beam size is diminished to 79 µm (H) × 14 µm (V) at *h*ν = 1600 eV, suggesting that the maximum of the beam size should be between 1000 and 1600 eV. In Fig. 5 ▸(*b*), beam sizes at 100 and 300 eV with the 300 lines mm^−1^ VLSG are also plotted by square symbols. Here, the higher *h*ν gives smaller widths in both directions. Although an exact origin of the photon-energy dependence has not been clarified yet, we suspect that heat load on the optical elements, especially the M1 mirror, should affect the beam size because of a deformation of the reflective surface of the mirror. The smaller beam size at 1600 eV compared with that at 1000 eV could be due to the lesser heat load because of a significant reduction of the brilliance of the undulator radiation above 1000 eV (Tsuchiya, 2015 ▸). More elaborate studies are required to assess the contribution of the heat effect on the beam size.

**Figure 6:**
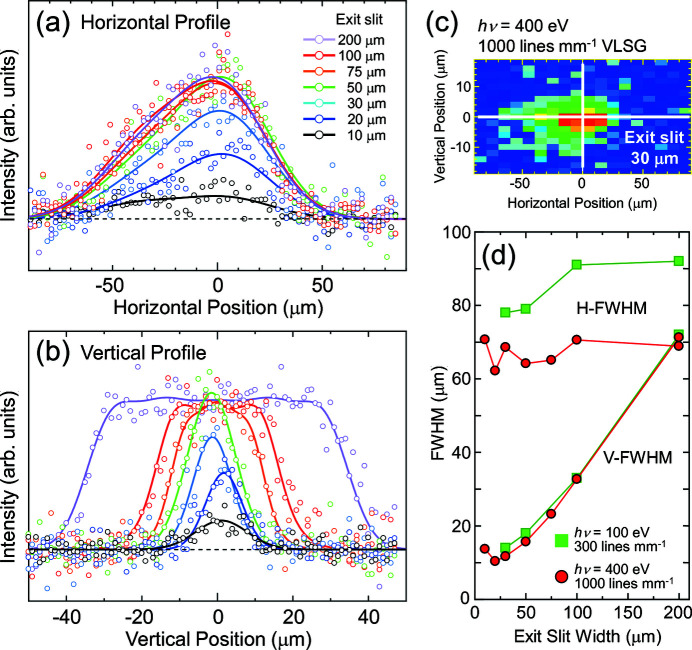
Exit-slit dependence of beam profiles in (*a*) the horizontal and (*b*) the vertical directions. The horizontal linear polarized light with energy of 400 eV was used. The beam profiles were acquired by 1D scans along the white lines in the panel (*c*), which shows a beam spot obtained by a two-dimensional scan. The open circles are the measured data and solid lines are results of least-squares fitting by single or multiple Gaussian functions. The H- and V-FWHMs are plotted in panel (*d*) for 400 and 100 eV light using circles and squares, respectively.

Figs. 6 ▸(*a*) and 6 ▸(*b*) show beam profiles of the horizontal and vertical directions measured at various exit-slit widths between 10 and 200 µm. The photon energy was 400 eV with the 1000 lines mm^−1^ VLSG. The beam profiles were obtained by one-dimensional (1D) scans at horizontal and vertical positions of 0 µm, which corresponds to the white lines in Fig. 6 ▸(*c*). The photon intensity monotonically enhances up to 50 µm with an increasing slit width along both horizontal and vertical directions. A further slit opening above 50 µm does not affect the horizontal profile, but the width in the vertical profile increases while keeping the maximum height. Circles in Fig. 6 ▸(*d*) indicate the estimated H- and V-FWHMs of the beam profile. The H-FWHM does not show a particular dependence on the slit width, having an average value of 67 (±5) µm. However, the V-FWHM of 71 µm at the slit width of 200 µm is almost linearly diminished to 16 µm by closing the slit down to 50 µm, and the diminishing rate is slowed from 50 to 20 µm. Since the vertical magnification of the M3 toroidal mirror is 1:0.4, the exit-slit-width dependence of the V-FWHM values almost follows the expected ones between 30 and 200 µm. For a slit width below 20 µm, the V-FWHM is not reduced further but seems to be saturated. We have also assessed the beam size at *h*ν = 100 eV with the 300 lines mm^−1^ VLSG and found that it has essentially the same trend, as indicated by squares in Fig. 6 ▸(*d*).

**Figure 7:**
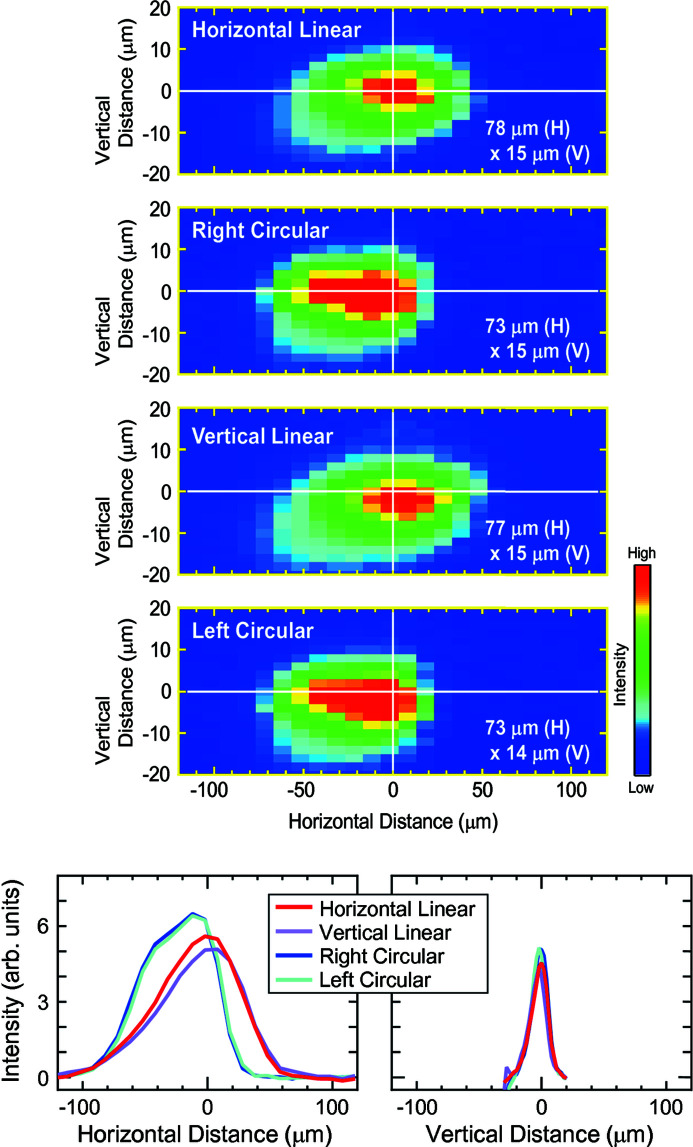
Light polarization dependence of the beam profiles (seen from the upstream side). The photon energy and the exit slit were set to 100 eV (300 lines mm^−1^ VLSG) and 30 µm, respectively. The measurements were performed in the order of the horizontal-linear polarization, right-circular polarization, vertical-linear polarization, and left-circular polarization. Integrated intensities along the vertical and horizontal axes are shown at the bottom. The origin of the vertical and horizontal axes corresponds to the maximum intensity in the integrated intensity curves of the beam with horizontal linear polarization.

Finally, we examined the light polarization dependence of the beam profile. Fig. 7 ▸ shows the profiles of the 100 eV beam (300 lines mm^−1^ VLSG) with (from top to bottom) horizontal linear, right-circular, vertical linear, and left-circular polarizations. By changing the polarization, although the beam height is almost unchanged with an error of less than 2 µm, the beam position is offset by about 20 µm in the horizontal direction between the linear and circular polarized light. Moreover, the horizontal size of the beam shows a polarization dependence: the linear polarized light has sizes of 77–78 µm, while the size of the circular polarized light is 73 µm, which corresponds to a ∼7% shrink. Two contributions are expected for the variations of the beam position and size in the horizontal direction: the first is a shift of the emission point of the synchrotron radiation, which may be caused by a slight deviation of actual undulator magnetic fields from the ideal ones, and the second is the effect of heat load on the beamline optics with a lesser load of the circular polarized light than the linear polarized light.

## 5. Demonstration of microscopic performance

To demonstrate the microscopic performance of the upgraded SES200 system at BL-13B, grid images were acquired by XAS and XPS. We used a square-mesh copper grid with 200 mesh (3 mm in diameter and 20 µm in thickness, EM Japan), whose bar width and opening were 35 and 90 µm, respectively. Since the as-loaded grid surface without any cleaning treatment was contaminated with carbon-containing species, C-related signals were observed with sufficient intensities in both XAS and XPS spectra. Therefore, we used the C signals to visualize the grid. For the microscopic XAS measurement, the photon energy was set at 285.2 eV (1000 lines mm^−1^ VLSG), which corresponded to the absorption peak in the C *K*-edge region. The focused beam was injected from the normal direction, and the total electron yield, *i.e.* the sample drain current, was monitored. For the microscopic XPS measurement, the C 1*s* peak at the binding energy of 286 eV was measured with *h*ν = 400 eV (1000 lines mm^−1^ VLSG). The C 1*s* spectra were acquired by a fixed mode with a pass energy of 150 eV. The incidence angle of the light was 65° (relative to the surface normal direction), and photoelectrons emitted around the surface normal were collected. The exit-slit width was 30 µm for the XAS and XPS measurements.

**Figure 8:**
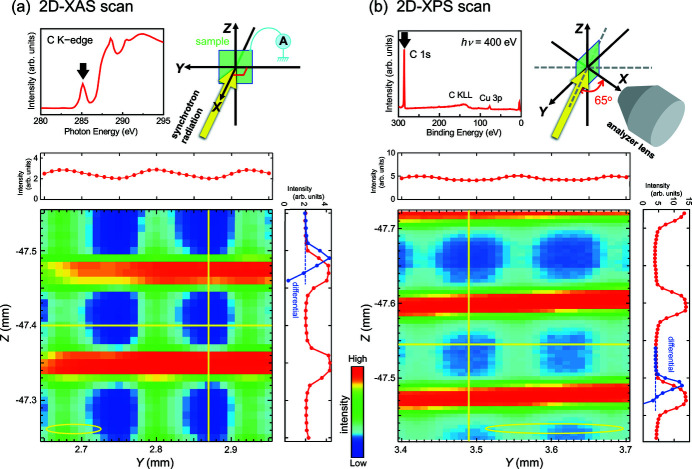
(*a*) 2D-XAS and (*b*) 2D-XPS intensity maps of a copper grid (200 mesh). The absorption intensity at the C *K*-edge (*h*ν = 285.2 eV) and the photoemission intensity of the C 1*s* peak acquired at *h*ν = 400 eV (indicated by arrows in the upper left panels showing the XAS and XPS spectra) were monitored to visualize grid images. The 1000 lines mm^−1^ VLSG and the exit-slit width of 30 µm were used. The incidence angles of the light were 0° (normal incidence) for the XAS scan and 65° for the XPS scans, as schematically drawn at the upper right parts. In the *Y*-axis scan, the measurements were made with a 10 µm step for both XAS and XPS. Along the *Z* axis, the measurement intervals were set to be 10 and 5 µm for the XAS and XPS scans, respectively. Ellipses drawn at the bottom of the 2D images represent the estimated sizes of the light irradiation areas: 73 µm (H) × 12 µm (V) for 2D-XAS and 184 µm (H) × 13 µm (V) for 2D-XPS. Intensity modulations along yellow horizontal and vertical lines are shown at the top and right side of each 2D image, respectively. A part of the differential curve of the intensity curve along the *Z* axis is shown by a blue line in each right-side panel.

Fig. 8 ▸ shows the intensity distribution of the C-related peaks of the grid in a 300 µm × 300 µm region. The two-dimensional (2D) XAS image was measured with a 10 µm step along the *Y* and *Z* axes, whereas the 2D-XPS image was acquired with 10 and 5 µm steps along the *Y* and *Z* axes, respectively. At first glance, the spatial resolution looks worse for the 2D-XAS image than for the 2D-XPS image. This is caused by using the different step sizes along the *Z* axis so that the number of pixels that form the XAS image is half of that of the XPS image. Namely, apparent degradation of the XAS image is artificial and is not essential. As we will show below, appropriate measurement conditions allow us to obtain the images with a spatial resolution that is determined by the beam size.

The grid images in Fig. 8 ▸ were visualized with a higher intensity of the horizontal bars compared with the vertical bars in both 2D-XAS and 2D-XPS scans. This intensity disparity between the horizontal and vertical grid bars reflects an elliptical shape of the focused beam: based on the observation in Fig. 5 ▸(*b*), FWHMs for the beam spots at *h*ν = 285.2 eV (2D-XAS) and at *h*ν = 400 eV (2D-XPS) are expected to be 73 µm (H) × 12 µm (V) and 78 µm (H) × 13 µm (V), respectively. For the XAS scan at normal incidence, the irradiation area of the beam is the same as the spot size. For the XPS scan, however, the horizontal size of the irradiation area is estimated to be broadened to 185 µm for the XPS scan because of the incidence angle of 65°. A weaker intensity modulation along the *Y* axis in the 2D-XPS image compared with the 2D-XAS image is because the horizontal extent of the irradiation area is larger in the XPS measurement than in the XAS measurement (185 µm versus 73 µm). To improve the spatial resolution in the horizontal direction, the H-FWHM of the beam must be diminished by cutting the horizontal spread of the beam using an aperture in the beamline, though the decrease in the photon intensity is inevitable.

Contrastingly, since the vertical extent of the beam is not affected by the incidence angle of the light, there is almost no difference in the intensity modulation between the 2D-XAS and 2D-XPS images along the *Z* axis. For both images, intensity curves along the *Z* axes have dull shapes of square waveforms (see panels on the right side of the grid images in Fig. 8 ▸). This is due to the small but finite V-FWHM of the focused beams (12 or 13 µm). Under the condition that the beam V-FWHM is smaller than the grid dimensions (35 µm of the bar width and 90 µm of the opening), the total spatial resolution in the vertical direction can be estimated from the observed intensity maps. This is because the slope of the rise and fall of the intensity curve along the *Z* axis reflects the beam broadening in the vertical direction. From peaks or dips in a differential curve of the intensity curve, the vertical spatial resolution is estimated to be 20 µm for 2D-XAS and 14 µm for 2D-XPS. The latter resolution is nearly comparable with the V-FWHM of the beam profile (13 µm), but the former is obviously larger than the V-FWHM (12 µm). We assume that the larger resolution in 2D-XAS is caused by an extrinsic/artificial effect of the coarse step interval in the *Z* axis: a 10 µm step for the 2D-XAS versus a 5 µm step for the 2D-XPS. When the XAS intensity profile along the *Z* axis was measured with a 3 µm step (the result is not shown), the spatial resolution is determined to be 11 µm, which is in good agreement with the beam V-FWHM (12 µm). For the vertical direction, this result indicates that a measurement step of less than 5 µm should be required to obtain 1D profiles or 2D maps of material surfaces with a spatial resolution that is comparable with the beam profile.

## 6. Summary

An upgrade and the present status of the VUV-SX beamline BL-13 and the end-station at branch B at the PF have been described. An APPLE-II EPU provides synchrotron radiation with linear, circular, and elliptical polarization. The maximum photon flux is >1 × 10^13^ photons s^−1^ at *h*ν ≃ 100 eV (300 lines mm^−1^ VLSG), and 10^11^ photons are available up to *h*ν = 1400 eV. Even at *h*ν = 2000 eV, a photon flux of approximately 3 × 10^9^ photons s^−1^ is available, and this is enough to perform XPS measurements.

The upgrade of the SES200 end-station system aims at microscopic XPS, XAS, and ARPES measurements. The toroidal post-mirror with *R*_t_ = 98.2 m and *R*_s_ = 0.0698 m, corresponding to the vertical magnification ratio of 1:1, was replaced by one with *R*_t_ = 43.0 m and *R*_s_ = 0.0399 m, having a ratio of 1:0.4. This replacement reduced the beam size to 35–44% in the vertical direction and 35–92% in the horizontal direction. The beam size is not affected much by the light polarization. However, it does depend on the photon energy and the line density of the VLSGs (300 versus 1000 lines mm^−1^). For the 1000 lines mm^−1^ VLSG, the V-FWHM and H-FWHM of the beam increase linearly from 12 to 15 µm (V-FWHM) and from 70 to 100 µm (H-FWHM) between 200 and 1000 eV, while the beam size of the 1600 eV light is slightly diminished. For the 300 lines mm^−1^ VLSG, the beam size at 300 eV is more focused than that at 100 eV.

For microscopic measurements and the beam profile assessment, a high-precision *XYZ* translator, a rotary feedthrough, and a newly developed sample goniometer have been installed in the SES200 system to manipulate samples with six degrees of freedom, *i.e.* *X*, *Y*, and *Z* translations and polar, azimuthal, and tilt rotations. The new sample manipulator system allows actual control of the sample position with submicrometre accuracy, which is enough to perform microscopic XPS, XAS, and ARPES measurements. Utilizing focused beams and a high-precision sample manipulation, 1D and 2D scans for XPS and XAS measurements of a copper grid with 200 mesh were performed. We obtained the XPS and XAS intensity maps by monitoring contaminant derived carbon signals. Our analysis of the intensity modulation reveals that microscopic measurements are possible with a spatial resolution based on the beam size.

Although the synchrotron radiation facility (the PF) and the electron energy analyzer (SES200) are not the most up-to-date, the upgraded BL-13B system, by introducing the APPLE-II EPU, the focusing mirror, and the high-precision manipulator, enables microscopic measurements to be carried out and, thus, is promising for cutting-edge research in the field of catalysis science, surface science, and industrial materials science.
